# Crash and disengagement data of autonomous vehicles on public roads in California

**DOI:** 10.1038/s41597-021-01083-7

**Published:** 2021-11-23

**Authors:** Amolika Sinha, Sai Chand, Vincent Vu, Huang Chen, Vinayak Dixit

**Affiliations:** 1 Research Centre for Integrated Transport Innovation, School of Civil & Environmental Engineering, The University of New South Wales, Sydney, Australia; 2 IAG Chair of Risk in Smart Cities, Sydney, Australia

**Keywords:** Civil engineering, Sustainability

## Abstract

Autonomous Vehicles (AVs) are being widely tested on public roads in several countries such as the USA, Canada, France, Germany, and Australia. For the transparent deployment of AVs in California, the California Department of Motor Vehicles (CA DMV) commissioned AV manufacturers to draft and publish reports on disengagements and crashes. These reports must be processed before any statistical analysis, which is cumbersome and time-consuming. Our dataset presents the processed disengagement data from 2014 to 2019, crash data till the 10^th^ of March 2020 and supplementary road network and land-use data extracted from OpenStreetMap. Primary data are manually assessed and converted into an easily processed format. Our processed data will be advantageous to the research community and enable accelerated research in this domain. For example, the data can be utilised to discern trends in disengagement, observe the distribution of disengagement causes, and investigate the contributory factors of the crashes. Such investigations can subsequently improve the reporting protocols and make policies and laws for the smooth deployment of this disruptive technology.

## Abstract (summary)

**Table:** 

Measurement(s)	autonomous vehicles • disengagement • crashes • reaction times
Technology Type(s)	manual entry • OpenStreetMap • Google Maps
Factor Type(s)	temporal interval
Sample Characteristic - Location	State of California

Machine-accessible metadata file describing the reported data: 10.6084/m9.figshare.15049227

## Background & Summary

Autonomous Vehicles (AVs) are being widely tested on public roads in several countries around the world. Although AVs are becoming more competent due to advancements in sensing and navigation technologies, safety stands as the biggest challenge in adopting this disruptive technology^1^. Currently, there are two major categories of “field” datasets that are publicly available; the crash and disengagement reports by the California Department of Motor Vehicles (CA DMV) and the extensive sensor (lidar and camera) datasets like KITTI^2^, Waymo^3^, etc. The latter category of datasets is proficient for accelerating research in computer vision, localisation, and behaviour cloning but lacks information about the critical instances (crash and disengagements). On the other hand, CA DMV disengagement and crash reports are the foremost publicly available datasets containing information about the critical instances. These critical events data are beneficial for laying a roadmap for the deployment of AVs, which includes reporting protocols, legal frameworks, and infrastructure maintenance plans. Table 1 presents the different categories of datasets used by researchers to evaluate the safety and performance of AVs.

**Table 1:** Different Categories of Datasets Available for Analysing AVs.

CA DMV data reports	Sensor data (KITTI, Waymo, etc.)	Microsimulation and driving simulator data
• Constitutes field data, and hence the system is exposed to unprecedented scenarios.	• Constitutes field data, and hence the system is exposed to unprecedented scenarios.	• Constitutes artificially generated or simulated data, and hence the system is not exposed to unprecedented scenarios.
• Includes the crash and disengagement reports.	• Includes sensor data (camera and lidar data).	• Includes trajectory information of AVs along with other vehicles.
• Contains information about the critical events (disengagements and crashes).	• Do not contain information about the critical events.	• Contains information about critical events (excluding the ones caused by perception error).

For the transparent deployment of AVs in California, the CA DMV commissioned AV manufacturers to draft and publish reports on disengagements and crashes. As a result, several researchers used the CA DMV reports to evaluate AVs’ safety-critical events. Most of the earlier studies focussed on discerning the trends in disengagements and crashes involving AVs^4–9^. Recently, a few studies have used these reports for developing statistical models. For example, one such study developed a nested logit model using three distinct outcomes: (1) no disengagement, (2) disengagement with no crash, and (3) disengagement with a crash^10^. The study also estimated endogenous switching models and deduced that disengagements lessen over time, and factors related to AV systems and other roadway participants elevate the tendency of disengagement without a crash. In another study, driving mode (autonomous vs manual mode), collision location, roadside parking protocol, rear-end collision, and one-way road were found to be significantly influencing crash severity^11^.

Another study developed fixed and random parameters binary logistic regression models to evaluate disengagement initiation^12^. The study emphasised that unfitting interpretations are possible if the random parameter approach is not used. Significant variables included location (highway or street), maturity of testing (months of testing), and cause (environmental, another road user, hardware or software discrepancy, and path planning discrepancy). The study also found that as the maturity of the testing increases by a month, the probability of automated disengagement increases by 0.014. Another study developed a Bayesian latent class model that identified six classes of collision patterns^13^. The authors pointed out the necessity of more advanced and robust collision narrative reporting for better analysis of the critical events. More recently, machine learning techniques were used to evaluate the crash severity of AVs^14^.

As evident, more complex models are being developed lately based on the CA DMV reports. However, these reports must be processed before conducting any statistical analysis, which is cumbersome and time-consuming. Furthermore, new crashes and disengagements are happening with more test vehicles on the road, and new data are appended regularly to the CA DMV dataset. The exclusion of the latest data used in the previous studies can result in premature findings^7^ and require constant scrutiny. Additionally, the advancement of this technology may change the currently observed trends^15^. Since the crash data are considered over a prolonged period, the time-varying explanatory variables may vary significantly. Neglecting latent within period variation may result in the loss of crucial explanatory variables. This loss of information by using discrete-time intervals can institute error in model estimation because of unobserved heterogeneity^16,17^, and hence studies using this data are required to be updated in the light of new information available.

In this context, we present the processed disengagement data from 2014 to 2019, crash data till the 10th of March 2020 and supplementary road network and land-use data of the AV testing locations in California. For the preparation of the current dataset, primary data in the form of crash and disengagement reports were requested from CA DMV. The primary data are extracted and organised into the processable format for potential reuse. Furthermore, OpenStreetMap and Google maps were used to extract supplementary data, which could be utilised to expand the research around AVs’ safety like in a previous study^11^, preparing guidelines and governance for AV deployment on public roads.

Specifically, the research community, government organisations, and public forums active in AV-related safety studies can benefit from this processed dataset. A comprehensive reporting protocol is necessary for the well-ordered deployment of AVs on public roads in any area. Henceforth, research can be conducted using our dataset towards identifying the shortcomings and fallibility of the reporting protocols used by CA DMV and assist government organisations around the world to devise better protocol systems for transparent deployment of AVs on public roads. For example, a limitation of the current CA DMV reports was pointed out by a previous study^4^, which stated to include the categories of disengagements in the reporting protocol by analysing the categorisation of disengagements into proposed macro and micro categories. Macro-level and spatial models of AV crashes can also be estimated using the supplementary data provided by our spatial database. They can be utilised to assess the hotspot crash/disengagement locations. Furthermore, the rate of disengagements can be affected by expanding the testing area as new and unfamiliar roads can generate new challenges for the AV brain. It is recommended to include testing area details in the reports. Also, new studies are advised to take into account the effect of spatial features. Eventually, managing a database that can be directly used without further processing or organisation could help accelerate the research and promote consistent terminologies, which is the contribution of this dataset. The processed data can bring consistency in the studies since different authors might use different appellations and can be readily imported to modelling and analysis software, thereby facilitating reproducible research.

## Methods

Our processed dataset contains disengagement data from 2014 to 2019 and crash data until the 10^th^ of March 2020. The disengagement primary data are reported by CA DMV at the end of every year, and the crash primary data are continuously updated. There are four kinds of data in our repository, viz. disengagement data, crash data, street network data and land use data. The procedure of their extraction is presented in the following sections.

### Disengagement and crash data

Primary data, i.e. disengagement and crash reports, were requested from CA DMV^18^. The reports are in pdf files and scanned copies of handwritten text. Authors manually scrutinised each report and converted them into excel and comma-separated files that can be processed by statistical tools like Stata, Nlogit and can be imported directly using scripting languages like Python or R for further analysis. A consistent data entry convention was discussed and developed considering different tools and scripting languages. The process of converting the reports into a processable format was time-consuming and cumbersome. More than three months was invested in going through reports and processing them. Automation of extraction of data from reports was infeasible since some manufacturers are submitting handwritten reports.

Additionally, some reports have subjective responses and hence required manual data entry and an expert’s elucidation. As a result, the data preparation required extensive manual hours investment. Furthermore, an extensive discussion was required to deduce the features that are not explicitly reported but can affect the crash, such as location characteristics (intersection, parking protocol and traffic light) and vehicle type. The latitude and longitude of every crash location were extracted from Google maps, and the “street view” feature of Google maps was used to discern location characteristics. The car model of the non-AVs involved in the crash mentioned in the reports was used to discern the “vehicle type” and resources like Wikipedia and manufacturers’ websites were used. Some of the key descriptive statistics of the crash data are presented in Table 2. Through the availability of this processed data, research groups worldwide could bypass these cumbersome steps and accelerate their research.

**Table 2:** Variable Definitions and Summary Statistics of Crashes from Our Dataset^22^.

Variable	Type	Definition	Summary statistics
Intersection	Dummy	=1 (if crash location was intersection); =0 (otherwise)	F (1) = 63%
Signal (signalised/ non-signalised intersection given that crash happened at an intersection)	Binary	=1 (with signals); =0 (without)	F (1) = 49%
Parking provision (Presence of parking at crash location)	Binary	=1 (with parking provision); =0 (without the provision of on-street parking)	F (1) = 56%
Mode (AV’s driving mode)	Binary	=1 (autonomous); =0 (conventional)	F (1) = 54%
Fault (Party responsible for the crash)	Binary	=1 (AV at fault); =0 (not at fault) The fault determination protocol is similar to the fault determination in the case of manual driven vehicles.	F (1) = 12%
AV status (Kinematics of AV)	Binary	=1 (moving); =0 (stopped)	F (1) = 57%
Other party’s vehicle status (Kinematics of non-AV)	Binary	=1 (moving); =0 (stopped)	F (1) = 95%
Road type	Dummy	=1 (street); =0 (otherwise)	F (1) = 69%
Vehicle type (Indicator of non-AV vehicle’s size)	Categorical	=1 (two-wheelers, i.e. bicycle, scooter); =2 (sub-compact/compact cars); these are identified as having an inside volume between 2.4m3 and 3.1m3 by combining cargo and passenger volume. =3 (mid-size cars) these are identified as having an interior volume between 3.1m3 and 3.4m3. =4 (trucks/buses).	F (1) = 12% F (2) = 41% F (3) = 36% F (4) = 12%
Collision type (Typology of collision)	Categorical	=1 (sideswipe); =2 (rear-end); =3 (others)	F (1) = 21.80% F (2) = 61.20% F (3) = 17%
Intersection Type (Type of intersection given that crash happened at an intersection)	Categorical	=0 (straight); =1 (Y-intersection); =2 (T-intersection); =3 (Cross-intersection); =4 (Complex intersection)	F(0) = 9% F(1) = 11% F(2) = 15% F(3) = 59% F(4) = 5%
Relative Velocity	Continuous	Relative velocity of the vehicles involved in the crash at the time of impact.	Reported only by a few manufacturers (176 out of 259 crashes reported relative speed)

### Street network and land use data

Python was used in the extraction of both street network and land use data. GeoPandas^19^, OSMnx^20^, and Pandas^21^ modules were used to assist with data manipulation. The geographical coordinates specified by the user (geocoded from crash data) are used to extract the road network (edges and nodes) using the OSMnx module. Custom filters are used as additional arguments to limit the results of the process (i.e., only returning edges and nodes, and not additional map features like pedestrian paths and building structures). Any missing features (due to incomplete data from OpenStreetMap) are assigned default values as appropriate (such as speed limits). Edges with multiple classifications are assigned only the highest classification (e.g., if an edge is both “primary” and “residential”, assigned only “primary” attribute). Any non-one-way roads are adjusted so that there are two edges for each of those roads (one for each travel direction). The street network data is in the form of shapefiles which can be opened using any geographic information system (GIS) tool such as QGIS or ArcGIS. Finally, the land-use data, in the form of points of interest (POI) such as restaurants, clinics, banks, etc. are also extracted from OpenStreetMap using OSMnx, by specifying the same coordinates as were used for network extraction. Extraction of spatial information took additional 20 hours.

## Data Records

This section discusses the organisation of the processed data in the *figshare* repository^22^. Table 3 outlines the structure of the repository. There are four folders in the repository.

**Table 3:** Data Repository Structure.

Folder Name	Description	
DMV Crash Report/	The folder contains one spreadsheet consisting of the crash information from original reports.	
DMV Disengagement Report/	The folder contains six spreadsheets. Details are provided in the Data Records section.	
Shape File/	Crash Data/	This folder contains spatial information about crash locations. (.qgz, .cpg, .dbf, .prj, .qpj, .shp, .shx files)
	San Francisco area 1/	This folder contains spatial information about road network in area 1. (.cpg, .dbf, .prj, .qpj, .shp, .shx, .csv files for edges and nodes)
	San Francisco area 2/	This folder contains spatial information about road network in area 2. (.cpg, .dbf, .prj, .qpj, .shp, .shx, .csv files for edges and nodes)
LandUseData/	poi_list Area 1 and poi_list Area 2 spreadsheets contain the land use data for Area 1 and Area 2, respectively.	

Note: Detailed descriptions can be found in the “ReadMe” files in repository^22^.

### DMV Crash Report (Secondary Data)

The folder includes a Microsoft Excel file “DMV Crash Data.xlsx”, which contains three sheets. The *Crash* datasheet contains the processed information from the original reports of CA DMV (primary data) and is manually inputted into this excel file. *README* sheet contains the variable definitions, and the *Variable* sheet contains the summary of the data.

### DMV Disengagement Report (Secondary Data)

The DMV Disengagement Report folder contains seven Microsoft Excel files, and their description is provided below.

- ○2014_DISENGAGEMENT, 2015_DISENGAGEMENT, 2016_DISENGAGEMENT, 2017_DISENGAGEMENT, 2018_DISENGAGEMENT and, 2019_DISENGAGEMENT
  These excel files contain the yearly disengagement data extracted from the CA DMV reports (primary data) in the years 2014–2019. In each of these files, there are several tabs, of which the first one is a “README” tab that provides a brief description of the corresponding excel file and the data layout. The second tab in these files provides the summary of disengagement data within each year, including the miles driven and the number of manual and automatic disengagements by each company in each month of that year. The rest of the tabs in the Excel files provide individual disengagement records (not always reported by all companies), including the reason for disengagement and in some cases, the reaction time to take over after disengagement. For example, the 2016_DISENGAGEMENT file has six other tabs where the manufacturers have recorded individual disengagements. Similarly, 2017_DISENGAGEMENT and 2019_DISENGAGEMENT files have eight and thirty-five additional tabs, respectively, that have information for individual manufacturers. The 2018_DISENGAGEMENT file has 35 additional tabs containing information of individual manufacturers (Apple has three subfiles, and UATC (Uber) has 2, due to their large dataset).
- ○COMBINED_DISENGAGEMENT_SUMMARY

This excel file is a combined summary of all the aforementioned excel files, where the file displays the monthly miles driven, number of automatic and manual disengagements reported by each company from 2014 to 2019.

### Land Use (Supplementary Data)

**Fig. 1:**
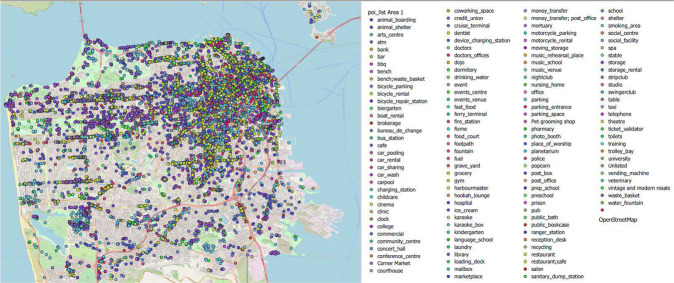
Land Use Data (Points of Interest). “Land use” is the term used to describe the human use of land. It represents the economic and cultural activities (e.g., agricultural, residential, industrial, mining, and recreational uses) that are practiced at a given place^24^. The land-use data, in the form of points of interest (POI) is presented in this figure. This figure is an illustration of land use data for area 1 (San Francisco). Each point in the figure represents the land use characteristics. The description of the coloured points\legends is presented in the list next to the figure.

**Fig. 2:**
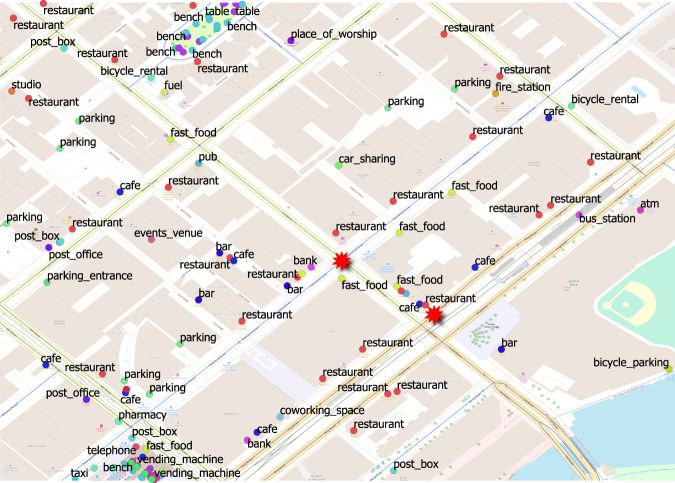
Land Use Data for a Sub-Area within Area 1. This figure represents the land use characteristics of a sub-area within Area 1. The different POIs can be better distinguished with the help of this figure.

This folder contains two comma-separated value (CSV) files; poi_List Area 1(San Francisco) and poi_List Area2 (San Jose), consisting of the information about the points of interest of these two areas, including the spatial coordinates. Figure 1 shows the map of Area 1 along with a legend for all the extracted POIs. Figure 2 represents a sub-area within Fig. 1 for better distinguishment of the different POIs.

### Street Network Data (Supplementary Data)

**Fig. 3:**
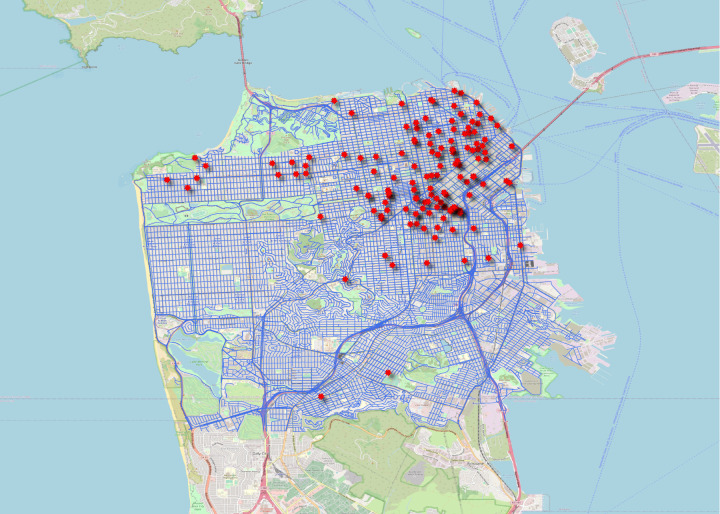
San Francisco (Area 1) Crash Locations Superimposed on the Road Network. The location (using latitude and longitude coordinates) of every crash is mapped into the testing area. Each point (red star) on this figure exhibits a crash location on the map for area 1, superimposed on the road network (the blue lines).

**Fig. 4:**
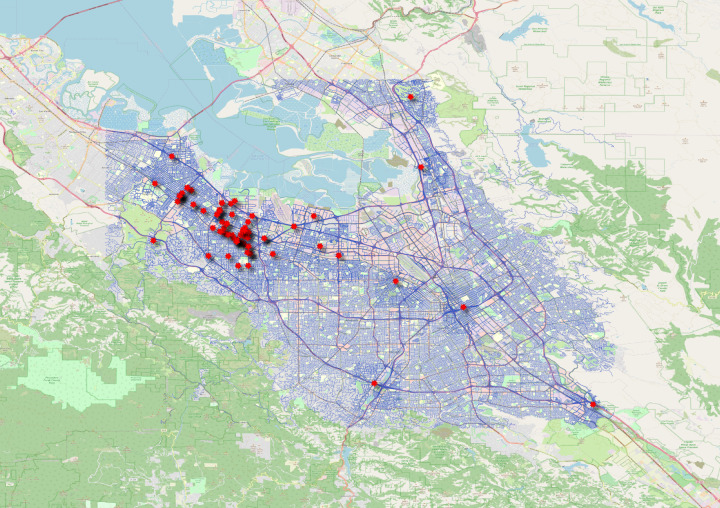
San Jose (Area 2) Crash Locations Superimposed on the Road Network. Like the Fig. 3, each point of Fig. 4 indicates a crash location on the map for area 2.

This folder contains two sub-folders: *Area1(San Francisco)*, *Area2(San Jose)* and *Crash Data*, which include the street network shapefiles (in the form of nodes and edges) for Area 1 and Area 2, respectively. The shapefile format is a geospatial vector data format for storing geometric location and associated attribute information relevant for geographic information system (GIS) software. Edges represent the physical streets, and the attribute table of edges includes information on the number of lanes, speed limit (in mph), the street name, type of road (freeway, primary, residential, etc.), and edge length. Nodes represent the intersection of edges, and their attribute table includes information on whether the node is a signalised or a priority sign intersection. Most of the crash records in the primary data provided by CA DMV did not include the spatial coordinates of the crash location. Instead, they include a description of the streets where the crash happened. Google Maps was used to geocode the text and convert it into spatial coordinates. The crash report provides a brief description of the crash. This includes the direction in which the vehicle is moving, approaching the intersection or placed at the intersection. Vehicle 1 movement provided information about the state of car (moving = 1, stationary = 0). The exact location of the crash was discerned using street information and a brief description. In case the report mentions “approaching intersection” or “at the intersection”, the geocode of intersection is provided. Otherwise, the midpoint of the street is geocoded. Figures 3 and 4 show the different crash locations superimposed on the road networks of Areas 1 and 2, respectively.

## Technical Validation

The reports are manually processed, annotated, and verified by the expertise. For instance, the reports have some subjective information, such as the seriousness of the injury and the location of the crash. First, one of the authors extracted the relevant data manually. Then the same task was performed by another author independently. It is important to note that these two authors did not have any direct connection with each other during the data extraction process. Once both the datasets were obtained, it was found that they were mostly consistent and had less than 2% contradicting data entries. Such records were then finally checked and corrected by a discussion among all the authors. At each of these steps, authors avoided using automated pipelines and used expert human intervention to register the implicit data.

Additionally, previously published studies were replicated with our processed crash and disengagement data, and the results were found to be consistent. The replicated results can be found in a recent study published by the authors^14^. The disengagement trends with appended data presented in our recently published study^14^ are coherent with a previous study^4^. The crash typology distribution and imbalance in injury outcome in our study^14^ are also coherent with another previous study^11^. These validation methods are discussed in detail below.

1. For the automated validation, data were imported to different software and scripting languages. The spatial data were imported to QGIS software and the csv files were imported to Stata and Python. Frequently used operations such as filtering, summarising, and grouping were performed. The importing and operation databases have been successful, and there are no missing data. Further, the ranges of each variable column were checked. For instance, there are four categories of “Vehicle type” defined. The summarisation of “Vehicle type” column was found to be consistent with the protocol used.
2. A further validation was explicitly done for spatial data. Spatial data points were plotted on the maps to verify that the spatial information/points fall within the test area. Figures 3 and 4 verify that all the points lie inside the testing area.
3. To validate our data, we compared our results with those from previous studies:
  Percentage of rear-end crashes
    A previous study^6^ reported that around 62% of crashes were rear-ended. Another study^11^ which analysed data until the year 2018, reported 63% of crashes as rear-ended. A more recently published study^14^ that used our dataset reported 61.78% (160 out of 259) rear-ended crashes. Consistent numbers reported corroborates validation of our dataset, as shown in Fig. 5. This figure can be compared with Vehicle #1 (AV) panel of Figure 7 of the previous study^6^ (refer to the following URL: 10.1371/journal.pone.0184952.g007).

**Fig. 5:**
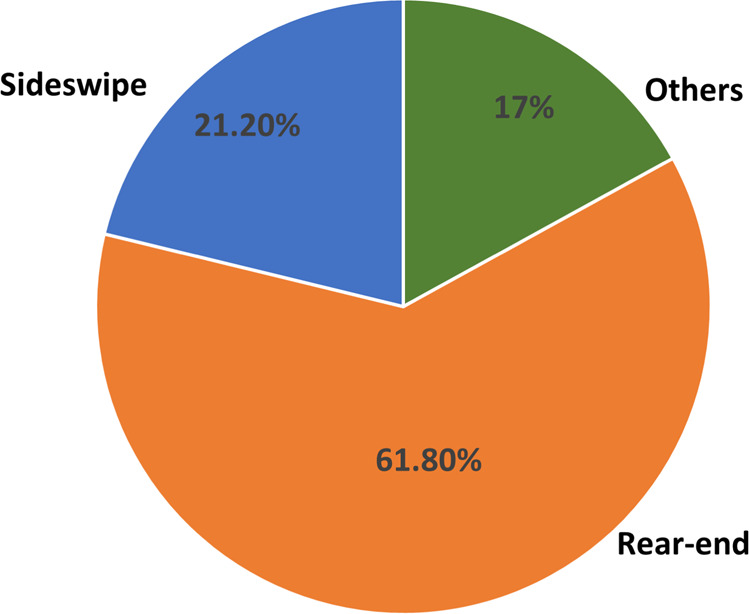
Crash type reported using our dataset. The percentage of rear-end crashes reported is 61.8%. A previous study^6^ reported that around 62% of crashes were rear-ended. Another study^11^ which analysed data until the year 2018, reported 63% of crashes as rear-ended. A more recently published study^14^ that used our dataset reported 61.78% (160 out of 259) rear-ended crashes. This figure can be compared with Vehicle #1 (AV) panel of Figure 7 of the previous study^6^ (refer to the URL: 10.1371/journal.pone.0184952.g007). These reported figures regarding the rear-end crashes were consistent with our dataset and thus reinforce the validity of our dataset.
  Distribution of crash severity
  A previous study^11^ reported 89% CAV involved crashes are property damage only (PDO) crashes. PDO crashes translate to no injury crashes. A similar percentage (85%) was reported with extended data (our dataset) in our recently published study^14^.
4. Lastly, the crash records reported by a previous study^6^ (refer to the following URL: 10.1371/journal.pone.0184952.t001) were compared with our dataset. Table 4 presents a detailed comparison. There were differences in the column names between the two datasets. For example, if our data reported Vehicle1 Status = 1 (Moving) and Vehicle1 Status = 1 (Moving), the study^22^ reported the Status of vehicles and relative direction = Moving/Moving. Furthermore, there are minor discrepancies in subjective columns, such as ‘injury reporting’ caused by the ambiguity of reporting protocol. Nevertheless, the interpretation of data was mostly consistent and thus, substantiate the validation of our dataset.

**Table 4:** Comparison between our dataset and a previous study^6^.

ID	Company	Date	Vehicle1 Status 1: Moving 0: Stopped (based on our data)	Vehicle2 Status 1: Moving 0: Stopped (based on our data)	Status of vehicles and relative direction M: Moving S: Stopped (as reported in a previous study^6^)	Vehicle1 Damage (based on our data)	AV Damage (as reported in a previous study^6^)	Injury Level (based on our data)	Injuries (as reported in a previous study^6^)
1	GM Cruise	2/16/2017	1	1	M/M	3	Minimal	1	No
2	Google	12/11/2016	1	1	M/M	3	Minor	0	No
3	Google	10/26/2016	1	1	M/M	3	Minor	0	No
4	Nissan	10/5/2016	1	1	M/M	1	Minor	0	No
5	Google	9/23/2016	1	1	M/M	5	Substantial	1	No
6	Google	9/14/2016	1	1	M/M	3	Minor	0	No
7	Google	9/7/2016	1	1	M/M	3	Minor	0	No
8	Google	9/2/2016	0	1	S/M	4	Moderate	0	No
9	Google	8/16/2016	0	1	S/M	4	Moderate	0	No
10	Google	8/8/2016	1	1	S/M	3	Minor	0	No
11	Google	7/15/2016	0	1	S/M	3	Minor	0	No
12	Google	5/4/2016	1	NA	M/NA	3	Minor	0	No
13	Google	4/28/2016	0	1	S/M	3	Minor	0	No
14	Google	4/7/2016	0	1	S/M	3	None	0	No
15	Google	2/14/2016	1	1	M/M	4	Only description	0	No
16	Cruise Automation	1/8/2016	0	1	M/S	3	Minor	0	No
17	Google	11/2/2015	0	1	S/M	3	Minor	0	No
18	Google	8/20/2015	1	1	M/M	3	Minor	1	Minor
19	Google	7/1/2015	0	1	S/M	3	Minor	1	Minor
20	Google	6/18/2015	0	1	S/M	3	Minor	0	No
21	Google	6/4/2015	0	1	S/M	2	None	0	No
22	Google	5/30/2015	0	1	S/M	3	Minor	0	No
23	Google	4/27/2015	0	1	S/M	2	None	0	No
24	Google	4/7/2015	1	1	M/M	3	Minimal	0	No
25	Google	2/26/2015	1	1	M/M	1	Only description	0	No
26	Delphi	10/14/2014	0	1	S/M	4	Only description	0	No

Note: This table presents a comparison of crash records reported in our dataset with that reported by a previous study^6^ (10.1371/journal.pone.0184952.t001). The columns from our dataset and the ones from the previous study are indicated in the column headers for easier comparison. There are minor discrepancies in subjective columns, such as ‘injury reporting’ which is caused by the ambiguity of reporting protocol. Nevertheless, the interpretation of data was mostly consistent.

## Limitations and Future Work

CA DMV reports are the best publicly available data containing information about critical events (crashes and disengagements) involving AVs. Nevertheless, the reports (and, therefore, our processed data) have few limitations. The reports (primary data) consist of a few fields like ‘damage severity’, which are assessed subjectively and may vary based on the assessor. Furthermore, the information of safety operation drivers is missing in the reports. Psychological attributes such as the risk attitude of the drivers could play a crucial part in discerning the details of safety operation^23^ but are not available in the reports. A previous study proposed a distinction between exogenous (factors outside the control of the driver and/or manufacturer, e.g., weather, infrastructure condition, outside traffic) and endogenous (factors can be acted upon) contributory factors of disengagement as an additional mandatory category in the reporting protocol^4^. However, this data lacks the difference within possible controllability of the factor, which can be crucial in discerning trends. Lastly, there is no data about conflicts. Authors believe that for a better understanding of the development of AVs, sensor data along with driver’s and road characteristics at the time of the crash should be made available to the government and researchers.

## Usage Notes

The excel sheets can be opened using any spreadsheet or workbook software. The shapefiles can be opened using any geographic information system tool such as QGIS or ArcGIS. The data can be directly imported to statistical or programming tools such as Stata, Nlogit and Python for performing analysis. We recommend that users review the sample reports in the database for further clarification.

The disengagement data can be utilised to discern the trends as performed in some previous studies^4,8^. Manufacturer-specific information like reaction time can be used for investigation as presented in a previous study^5^. The crash data can be utilised in two significant ways. First, descriptive statistics and identification of trends as done by some past studies^6^. Second, crash data can be used for exploratory investigation as performed by a few studies^11,13^. New fields are appended in the reports, and auxiliary data (spatial data, vehicle type etc.) can be utilised to develop more comprehensive models.

## Data Availability

Primary data (crash and disengagement data) extraction does not utilise any automated pipelines or scripts due to implicit and subjective data in the original reports. Experts manually analysed each report to register the data into processable formats. GeoPandas^19^, OSMnx^20^, and Pandas^21^ Python custom modules are used for the extraction of secondary data. Code documentation for GeoPandas (https://github.com/geopandas/geopandas) and OSMnx (https://github.com/gboeing/osmnx) can be found in the corresponding GitHub repositories. A more elaborative procedure is explained in the Methods section.
